# Technology-Mediated Enrichment in Aged Care: Survey and Interview Study

**DOI:** 10.2196/31162

**Published:** 2022-04-12

**Authors:** Jenny Waycott, Wei Zhao, Ryan M Kelly, Elena Robertson

**Affiliations:** 1 School of Computing and Information Systems The University of Melbourne Melbourne Australia; 2 No to Violence Melbourne Australia

**Keywords:** aged care, older adults, technology, social enrichment, virtual reality, robots, videoconferencing, care providers

## Abstract

**Background:**

Digital technologies such as virtual reality (VR), humanoid robots, and digital companion pets have the potential to provide social and emotional enrichment for people living in aged care. However, there is currently limited knowledge about how technologies are being used to provide enrichment, what benefits they provide, and what challenges arise when deploying these technologies in aged care settings.

**Objective:**

This study aims to investigate how digital technologies are being used for social and emotional enrichment in the Australian aged care industry and identify the benefits and challenges of using technology for enrichment in aged care.

**Methods:**

A web-based survey (N=20) was distributed among people working in the Australian aged care sector. The survey collected information about the types of technologies being deployed and their perceived value. The survey was followed by semistructured interviews (N=12) with aged care workers and technology developers to investigate their experiences of deploying technologies with older adults living in aged care. Survey data were analyzed using summary descriptive statistics and categorizing open-ended text responses. Interview data were analyzed using reflexive thematic analysis.

**Results:**

The survey revealed that a range of commercial technologies, such as VR, tablet devices, and mobile phones, are being used in aged care to support social activities and provide entertainment. Respondents had differing views about the value of emerging technologies, such as VR, social robots, and robot pets, but were more united in their views about the value of videoconferencing. Interviews revealed 4 types of technology-mediated enrichment experiences: enhancing social engagement, virtually leaving the care home, reconnecting with personal interests, and providing entertainment and distraction. Our analysis identified 5 barriers: resource constraints, the need to select appropriate devices and apps, client challenges, limited staff and organizational support, and family resistance.

**Conclusions:**

This study demonstrates that technologies can be used in aged care to create personally meaningful enrichment experiences for aged care clients. To maximize the effectiveness of technology-mediated enrichment, we argue that a person-centered care approach is crucial. Although enrichment experiences can be created using available technologies, they must be carefully selected and co-deployed with aged care clients. However, significant changes may be required within organizations to allow caregivers to facilitate individual technology-based activities for enrichment.

## Introduction

### Background

For many of the people now living into advanced old age, opportunities to engage in social, creative, or fun activities may diminish because of mobility constraints and decreasing social networks [1]. It can be especially challenging for those who move into institutional care homes to stay socially connected and engaged. Residential care homes provide 24-hour care and monitoring; however, they can be lonely places to live [2,3]. There may be few opportunities for aged care clients to leave the care home to socialize or engage in activities that provide them with joy and enrichment.

Psychosocial care, attending to people’s needs for social connectedness and emotional enrichment, is an important component of aged care service provision [4]. In Australia, where this study was conducted, this is provided through both residential care, for people living in care homes, and community-based care, for people living independently. In both types of care settings, social enrichment is often provided through a program of structured activities, which might include group games such as bingo, exercise, and music [5,6]. Many organizations are now incorporating technology-based activities into their social programs. These initiatives are often led by the care organizations [7]. There is also a large body of research evaluating trials of technologies used for social well-being in aged care, including robot pets for companionship and comfort [8], social robots for entertainment [9,10], videoconferencing and social networking tools for communication [11], video games for playful interactions [12], and virtual reality (VR) for reminiscence [13]. Communication technologies can be valuable for expanding older adults’ social lives, helping people who are otherwise alone feel a sense of connection with the world [14-17].

Researchers have noted, however, that not all older adults will gain benefit from technology-based social interventions [18]. There must be a good fit between the technologies being used (and the activities they are used for) and the needs of people being supported. Achieving this alignment requires care and attention from those responsible for introducing technology [19,20]. In residential aged care in Australia, this includes lifestyle coordinators who are responsible for running activity programs and technology vendors who are sometimes responsible for introducing technology into aged care.

Current research on the use of emerging technologies by older adults typically involves trials of specific technologies, focusing on the health and well-being benefits for clients [8,21]. These studies provide useful insights into the potential benefits of new technologies to enrich the lives of older adults; however, they do not provide a broader view of the issues faced during the process of deploying new technologies in aged care. However, past research has identified significant barriers to the successful implementation of emerging technologies (eg, social robots) in care settings [22-24], including technical problems [22], negative preconceptions about new technology [22], and a lack of acceptance from end users [24]. Care staff may need to invest additional time and effort to overcome these barriers, placing further burdens on their already busy schedules. This is particularly challenging in the Australian aged care system, where a Royal Commission recently revealed significant neglect partially because of underresourcing and insufficient staff training.

Given that residential aged care is a complex and sensitive setting, there is a risk that introducing new technologies may cause harm. Therefore, there is a need to understand and carefully manage the opportunities and challenges that occur when technologies are used to support people in aged care. Gaining an in-depth understanding of what works and does not work will help inform future good practice in this sensitive setting.

### Objectives

This study aims to understand how technologies are being used to enrich the lives of older adults in aged care and identify lessons for good practice in this area. This study focused on understanding the experiences of people responsible for introducing and facilitating technologies in aged care settings. This includes those who work in aged care, such as personal care assistants, diversional therapists, and lifestyle coordinators, along with external technology vendors and providers who introduce and deploy technology in aged care.

The study focused on the following questions:

1. What technologies are being used to provide social and emotional enrichment in aged care?
2. How do staff value the different technologies used for enrichment in aged care?
3. What kinds of enrichment experiences are enabled when introducing technology-based activities in aged care?
4. What challenges or barriers need to be overcome when using technologies for social and emotional enrichment in aged care?

## Methods

### Ethics Approval

All procedures were approved by the University of Melbourne Human Research Ethics Committee (ID 1851239.1). Survey respondents read a plain language statement about the study, which included information about the length of time required, anonymity of responses, and data management. Respondents had to provide consent before proceeding with the survey. Interviewees were provided with a copy of the plain language statement and signed a consent form before proceeding with the interview.

### Data Collection

#### Overview

This study involved a web-based survey targeting aged care staff and technology providers, comprising a combination of Likert scale and open text responses. The survey was designed to obtain information about the types of technologies being used for enrichment in the Australian aged care system. We also conducted semistructured interviews to gain an in-depth understanding of the experiences of aged care and technology providers using technology in aged care.

#### Web-Based Survey

The survey was designed using the SurveyMonkey tool and tested for length and clarity by the research team. It had 3 sets of questions (Multimedia Appendix 1). The first set of questions asked about background information, such as the respondents’ role in aged care. The second set asked respondents about their own experiences of deploying technologies in aged care using the question, “What type of technology have you used in aged care for social or activity purposes?” The question specified *social or activity purposes* to guide respondents to nominate technologies used as part of the activity programs in aged care; that is, to provide social and emotional enrichment, in contrast to technologies used to support medical care. Participants were asked to describe the technologies and why they were using them in free text boxes.

In the third set of questions, respondents used Likert scales ranging from 1 (not at all valuable) to 5 (highly valuable) to rate the perceived value of the following 6 technologies: VR, robot pets, social robots, social networking tools or systems, videoconferencing tools, and digital storytelling apps. These technologies were chosen because of considerable interest in their use in aged care [21,25-28]. Respondents had the option to select *not applicable* if they did not know enough about the technology to make a judgment.

#### Interviews

A total of 12 interviews were conducted, 9 (75%) via phone or internet and 3 (25%) in person. Interviewees were asked to provide details about their experiences of deploying technologies in aged care and their views on the benefits and challenges involved in using different technologies to enrich the lives of aged care clients (Multimedia Appendices 2 and 3). The interviews focused on technologies used to support psychosocial caregiving rather than those used to support instrumental care, such as sensors for fall monitoring. All interviews were audio recorded and then transcribed verbatim for subsequent analysis.

### Participants

#### Survey Respondents

The survey was openly distributed to aged care and technology providers throughout Australia in late 2018 and republicized in late 2019. It was distributed via the researchers’ professional networks, notices in relevant email lists and industry publications, and social media platforms (Twitter and LinkedIn). The survey was open (not password protected). No incentives were offered for responding to the survey.

We received 20 complete responses to the web-based survey. Table 1 shows the different types of aged care provided by the respondents. Respondents worked in various roles, including as activity coordinators and project managers, and in design innovation, community engagement, and operational excellence. Other respondents included nurses and consultant geriatricians working in hospital care units as well as developers and managers working for technology vendors.

**Table 1:** Web-based survey respondents: type of aged care organization (N=20).

Organization	Values, n (%)
Residential aged care	9 (45)
Mixed residential and home-based aged care	4 (20)
Hospital care units	3 (15)
IT^a^ providers	4 (20)

^a^IT: information technology.

#### Interviewees

We conducted a total of 12 interviews, and 6 (50%) interviewees completed the survey and agreed to participate in the follow-up interview. We contacted other potential interviewees directly and identified them through their professional networks. We used purposive sampling to ensure that we included people with expertise in using technology for enrichment in aged care. All the interviews were conducted between October 2018 and December 2019. Table 2 provides more details about the interviewees, among whom 67% (8/12) worked in aged care facilities as care staff, lifestyle team members, or managers. The remaining 33% (4/12) of the interviewees were technology developers and vendors responsible for introducing technology into aged care settings. All had the experience of introducing new technologies in aged care for providing social and emotional enrichment for aged care clients.

**Table 2:** Interview participants.

Pseudonym	Perspective	Survey completed
Alan	IT^a^ company (founder)	No
Barry	Care provider (service or project manager)	No
Claire	Care provider (pastoral care)	No
Del	Care provider or IT (innovation manager)	Yes
Eric	Care provider or IT (project officer)	Yes
Frank	Care provider (volunteer)	Yes
Graham	IT company (founder)	No
Helen	Care provider (funding manager)	Yes
Ian	IT company (founder)	Yes
Jacquie	Care provider (lifestyle manager)	No
Ken	Care provider (CEO^b^)	Yes
Larry	IT company (founder)	No

^a^IT: information technology.

^b^CEO: chief executive officer.

### Data Analysis

Quantifiable survey responses were analyzed using summary counts and descriptive statistics. The open-ended survey responses and interview transcripts were analyzed using reflexive thematic analysis [29]. The data were coded by WZ and JW using an inductive approach. WZ identified initial themes, which included 8 themes under the *benefits* category and 15 under the *challenges* category. These were documented in a written report for discussion among the researchers. JW conducted the next stage of the analysis, refining and combining the themes to identify a final set of 9 themes, 4 that aligned with benefits, described below as technology-mediated enrichment experiences. The 15 challenges were recategorized into 5 overarching themes associated with key barriers to the effective implementation of technology-based enrichment experiences in aged care.

## Results

### Web-Based Survey

#### Types of Technologies Used

VR was the most common technology used by the survey respondents (13/20, 65% of respondents). Other popular technologies included computer or video games (10/20, 50% of respondents) and social networking systems (8/20, 40% of respondents). Commercial VR products such as Samsung Gear, Google Daydream, HTC Vive, and Oculus VR systems had been used for a variety of purposes, including virtual tours, reminiscence, entertainment, pain distraction, and staff training. Computer or video games ranged from digital card games to exergames. Social networking tools such as Facebook were used for social connectedness, whereas some respondents described bespoke apps that were designed for social connectedness.

A total of 30% (6/20) of the respondents said they were using *tablets and mobile phones*, and 25% (5/20) were using *CDs, DVDs, radios, and televisions* for enrichment. Tablets and mobile phones were used for facilitating video calls through apps, such as Skype and Facetime, and for providing older people with personalized music, games, audiobooks, and video browsing experiences. CDs, DVDs, radios, and televisions provided similar functions. A survey respondent described using a customized television that displays only old-time music, programs, and commercials, aiming to connect people with their old memories.

A total of 25% (5/20) of the respondents used social or companion robots in aged care. These included robot pets, such as Paro, the seal and Hasbro toy animals, which were primarily used for comfort, diversion, and entertainment purposes.

#### Perceived Value of Different Technologies

**Figure 1:**
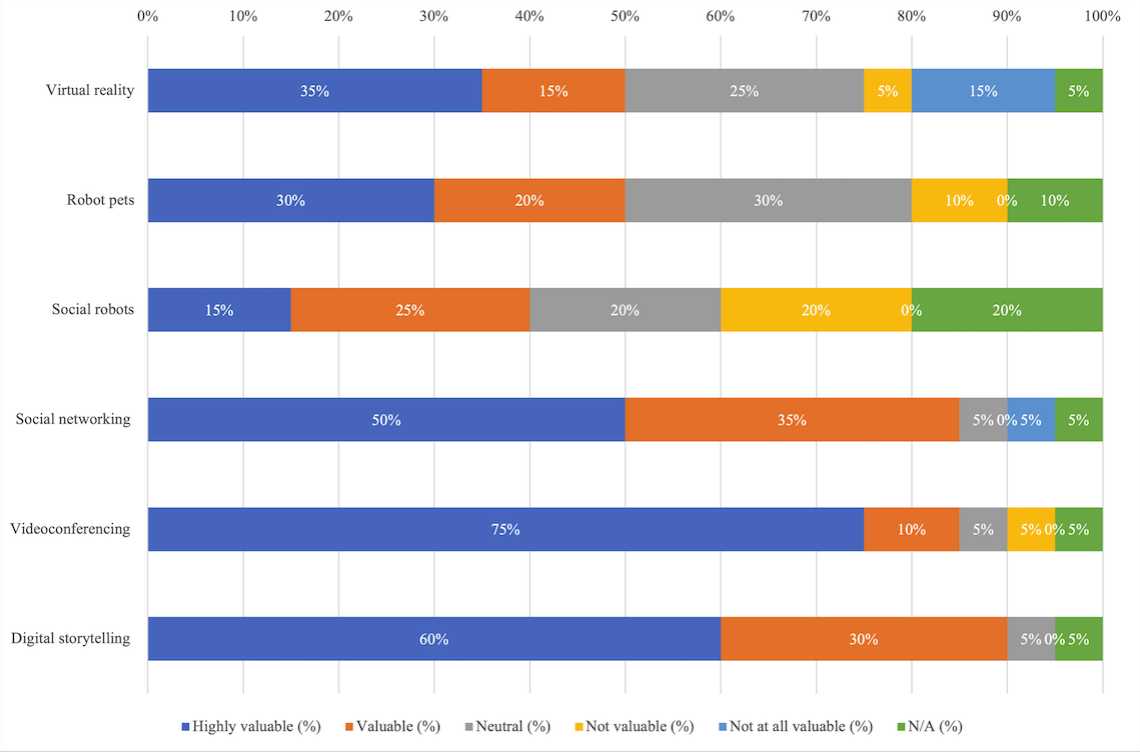
Distribution of respondents’ perceived value of different technologies. N/A: not applicable.

Figure 1 shows the percentage of survey participants who perceived different technologies as highly valuable, valuable, neutral, not valuable, or not at all valuable. “Not applicable” denotes that they did not know enough about the technology to make a judgment. Notably, VR, robot pets, and social robots, which could be considered the most innovative of the technologies listed, had the highest variation in perceived value.

At the end of this block of Likert scale ratings, respondents were asked if they had any comments about why the technologies listed were or were not valuable for use in aged care. Earlier in the survey, they were asked to comment on the benefits and challenges they had faced when using technology in aged care. We examined these responses together to identify the perceptions about specific technologies. Where respondents' comments are included below, they are identified as S1 to S20 to indicate which survey respondent made this comment. Regarding the use of VR, although some respondents (6/20, 30%) found VR to be useful for virtual travel, there were also concerns about the high cost and frequent need for troubleshooting when using VR (3/20, 15%), the difficulty of using it for people with dementia (2/20, 10%), potential for discomfort such as headache and dizziness (3/20, 30%), the burden on staff time and need for staff training (3/20, 30%), and perceived resistance from clients (2/20, 10%). For example, a survey respondent said that a client had said, “I don’t want to put electricity to my head” (S18).

Some respondents (6/20, 30%) believed that robot pets could provide beneficial outcomes for people with dementia or those experiencing loneliness. Others expressed reservations about their value; for example, “Some that I’ve seen have been a bit spooky and border on child-like toys” (survey respondent [S] 8) and “[They] are just a novelty and gimmick long term as they do not foster real person-to-personal connection” (S13). Social robots that talk and provide entertainment [10] were valued for their potential to address social isolation but were also seen to lack *real person-to-person connection* (S13). A respondent noted that commercial smart speakers could provide similar benefits and were preferable over specialized robots:

At the moment, Google Home and Alexa can give some of the same benefits (company, feedback) that a social robot could. I think the social robot would have to offer something significantly enhanced in order to differentiate it.

S8

Videoconferencing tools received the most positive perceived value: out of 20, a total of 17 (85%) believed them to be valuable or highly valuable and 1 (5%) respondent expressed concern that it was difficult for clients with severe dementia to use videoconferencing tools, even with the help of staff. Similarly, we noticed a positive perception of values for social networking tools and digital storytelling apps. One respondent commented that social networking tools are useful for “allowing elders to connect with remote family members in dynamic and rich ways,” and other apps, for example, audio books and screen readers, are valuable “for those who cannot physically read words in a book, or even physically turn pages” (S13).

### Interviews

#### Technology-Mediated Enrichment Experiences

A thematic analysis of interview data identified four kinds of technology-mediated enrichment experiences: (1) enhancing social engagement, (2) virtually leaving the care home, (3) reconnecting with personal interests, and (4) providing entertainment and distraction.

##### Enhancing Social Engagement

Many interviewees discussed the social benefits that technology could provide for aged care clients. In particular, videoconferencing tools connect people living in residential aged care with loved ones who may not be able to visit them regularly. Claire captured the power of this connection:

I remember one lady who's actually just turned 101 last week. She is in Sydney while her daughter is in Melbourne. We had the iPad and did the Skype calls with her daughter. It’s so beautiful when they say goodbye to each other. She gets the iPad, and she kisses the iPad, and goes “love you [kiss].” It is so beautiful to see that interaction and for the daughter. She’s just so happy.

Claire

In addition to one-on-one communication, videoconferencing tools enabled clients to participate in family events. Claire shared another compelling example: a client who was a family matriarch who had always previously been involved in family events was devastated to learn that she was not attending her grandson’s wedding. In response, Claire suggested that they use Skype to “bring the wedding to you here.” This example shows the care and creativity required to create a meaningful connection for a person living in aged care:

Over the following three months we prepared this whole gathering where we would Skype the wedding, bring it here, and she agreed that all of the residents would be invited to the wedding...So we set up the whole place as a high tea, and we skyped in and...it was really really beautiful and then after the wedding was finished we turned it off...We then reminisced. Everybody started talking about wedding experiences, their own weddings, other people’s wedding, while we had this lovely high tea.

Claire

This story by Claire illustrates how technologies can be used to not only connect residents to the outside world but also facilitate conversation within the care home, suggesting that technology-mediated connections can provide multiple social benefits. Other interviewees described how some technology-mediated activities provided *talking points*; clients would share their personal experiences with caregivers, family members, or other residents after taking part in a new activity. This was particularly apparent for interviewees who used VR to enable clients to virtually *travel* to new places or revisit childhood hometowns:

VR has an ability to open up the mind in a way like nothing else I’ve seen. When [the headset] comes off, I then go back and have them share their memories of that place. It might be their brothers, their sisters, their parents, where they went to school, where they played, what it was like growing up, and they start to share the stories of their life. And they may go from a very dormant non-communicative state to actually having a full-on conversation for the first time in a long time. And that really helps the connection with families as well.

Frank

Interviewees emphasized that technologies should serve as a medium for promoting social interactions among people rather than as a stand-in for social interaction. Technologies such as social robots or smart speakers could potentially engage in conversations with users, thereby appearing to address people’s experience of loneliness and isolation. However, interviewees were generally not supportive of this concept, preferring to use technology that connected people to each other rather than to a device. When asked to describe what the *ideal technology* for older adults would look like, Alan said the following:

It would involve interacting with people rather than with an artificial intelligence, but it might have an artificial intelligence in it. I think it would be an active thing that involves the older adult having to do something...doing something important as distinct from just being entertained...Something meaningful. And ideally with people.

Similarly, Claire said the following:

When we talk about having robots in care [and] all these games and stuff, that’s all very well but it will never ever be the same in terms of meaning making [and] connection...They don’t want to be shoved in a corner somewhere and think it’s all over. It’s about staying connected. So whatever it takes to keep people connected, that’s what we do. That’s what the technology is good for.

##### Leaving the Care Home

Using technology to connect residents to the outside world provided benefits beyond social engagement. Interviewees described instances in which using new technologies gave clients something to look forward to, helped them access places they had been to in the past or would like to visit, and opened their world to new experiences. Immersive VR was particularly useful in realizing this benefit:

We had a lady who was in the [VR] session. She didn't speak any English. And she was 99 years old. And she loved it. She was at a stage where probably everyone told her you're never going to go anywhere, see anywhere again. So the idea of them being immersed in places like the Aurora Borealis, Egypt or the Middle East, the States. The world that just opened for that had been closed off.

Larry

The interviewees shared examples of using other tools such as YouTube or Google Earth to help residents stay connected with the outside world. Claire shared 1 example in which she helped a resident to go home using technology. Through this process, she gained opportunities for conversation, which helped alleviate her client’s distress:

I was asked to see her and she was crying and crying, just inconsolable, and...she kept on saying “I wanna go home, I wanna go home...”And I actually thought, “what am I going to do?” I do a lot of bereavement counselling and I thought, “oh, this is going to be really hard.” And I said to her, “tell me, where do you live? What’s your address?” And all I had was my phone. I put her on my phone on Google Earth, on satellite view. I said, “Let’s have a look where you live” and suddenly the place came up and she stopped crying! And she sat up and said, “No, my place has changed. We painted the garage door, and we chopped that tree down.” So obviously the photo was a little bit out of date, and it prompted this amazing conversation.

Claire

A key constraint for people living in residential aged care is that they may have few opportunities to experience life outside the care home. For Barry, being able to be transported to another place would be an ideal way for technologies to provide enrichment:

If we could have something that could “beam me up scotty” and take them to places that they have enjoyed in the past, that would be a technology that residents and people would certainly enjoy.

Furthermore, within a care home, people’s lives may be overly controlled and structured. Offering new experiences and the chance to virtually leave the care home could provide an antidote to the lack of control people have when living in residential care homes. For Del, the greatest benefit that technologies provide is “the ability to bring a different experience...it adds to a culture of greater flexibility rather than control or paternalism.”

##### Reconnecting With Personal Interests

Interviewees spoke at length about the value of using technology to connect people in aged care with their personal interests and passions. They noted that this benefit was evident when deploying technology one on one compared with conducting group activities. Interviewees shared stories about using VR or YouTube videos to help aged care clients rediscover their personal interests:

I had the privilege of working with a guy who was turning 100. He was a car fanatic. When asked about his dream, he said, “I would love to have been in one of the leading car categories in the world,” and he said “but today I would love to be able to sit in a Formula 1 race car.” So I went onto VR, I found a Formula 1 race car, I put the goggles on him, and he did a lap at the track in Germany, hollering at the top of his voice, whooping and hollering, having such a ball. And it’s something that he still talks about with his family, because he got to live a dream.

Frank

This story further emphasizes the unique capability of VR to provide a sense of being transported to a time or place outside of the care home. It also highlights how a joyful experience emerged, because the activity was designed to respond to the client’s past interests. This opportunity to realize a long-held dream appeared to create a sense of elation, which may have been momentary but would nevertheless have been valuable for an aged care client who may normally have few opportunities to experience such joy in his day-to-day life.

Another interviewee who ran a technology company that introduced information technology (IT) solutions for aged care described how it was important to identify people’s personal interests to find opportunities to use technology in meaningful ways. The interviewee described a client who was able to reconnect with past interests by watching YouTube videos:

There was a gentleman that’d been part of this Isle of Man race, a motorbike race in the 50s or 60s. We quickly went to YouTube, found the race, and the guy was able to re-live that race. And that’s not complex, but it takes conversation and trying to understand what’s possible. Some people are interested in games, some people are interested in learning. Some people are just interested in photos of the people they love, or Facetime or Zoom or whatever.

Graham

When asked about the ideal technology-based enrichment activity in aged care, Del pointed out the need to offer a range of experiences to cater to individual client needs:

It would be a smorgasbord. There would be a range of things for people to interact with...Choice and agency is really important, so your ability to choose a range of scenes, your ability to choose a range of experiences...

##### Providing Entertainment and Distraction

In addition to providing joy by reconnecting residents to their personal interests, technology-based activities also provided general entertainment and distraction. This was considered particularly valuable for those living with dementia, with technology-based activities distracting clients from the psychological and behavioral symptoms of dementia, including agitation, distress, and wandering. This benefit was particularly apparent when using robot pets and VR. For example, an interviewee noted the utility of Paro, the seal, a therapeutic robot, for calming and entertaining residents:

I think the benefits of the PARO seal, it gives that response, and it keeps residents happy, and if they wander it actually settles them down and calms them down. They couldn't stop her [one resident] wandering, but you give her the Seal and it just calms her down, and you get that complete, I'll say peace of mind that you don't have to worry that she's off trying to get out of the building.

Ken

For Helen, technology had the potential to help manage some of the more challenging behaviors associated with dementia, including violence. She noted that music, in particular, could be calming and that aged care homes should provide pleasant activities to give people joy and comfort at the end of life:

Wouldn’t you want something nice and bright and something around you when, you know, it’s the end of your life? And probably music is - anything with music is so calming and soothing for anybody, whether you’re young or old. So that sort of this could be helpful in the technology side.

Helen

Even simple activities, such as using an iPad to provide personal access to television programs, could give people an opportunity to escape discomfort. Eric shared an unusual example:

We’ve been using Snapchat with residents as an activity. They really enjoy playing with the Snapchat filters and seeing what they can do...They’re just taking photos with the iPad with Snapchat on it and sharing it around the facility.

Eric

This example shows that there can be value in using technology for playfulness and entertainment in aged care. The interviews demonstrated that technology-based activities can provide numerous ways of enriching the lives of older adults in aged care. Using technology effectively in aged care, however, involves managing many challenges and overcoming barriers, as discussed next.

#### Barriers to Technology-Mediated Enrichment

Our analysis identified five key challenges and barriers encountered by interviewees when introducing technologies for enrichment in aged care settings: (1) resource constraints, (2) selecting appropriate devices and apps, (3) client challenges, (4) limited organizational and staff support, and (5) resistance from families.

##### Resource Constraints

Participants described a range of constraints that limited their deployment of technologies in aged care, including funding, staff numbers, and workload. Financial constraints associated with business decision-making specifically affect the procurement of technology products and upkeep costs. According to Alan, aged care organizations focus on the following:

By and large [they are] focused on their profit and loss statements regardless of whether they're for profit or non-profit organisations. You know there's this kind of concept that non-profits are...trying to do things for love which is sometimes true but mostly they have budgets too and people who work there want to get paid. So the technologies that I've seen getting introduced into aged care environments mostly only work if they’re actually improving the bottom line of the organisation.

An interviewee commented on the disparity among regions, noting that aged care organizations in regional areas struggled more with funding issues, which led to difficulties in providing the infrastructure to support technology use in aged care:

City facilities are going to have more than what country facilities have. The sad part about being in the country is you’re a forgotten race really and...you’re not big enough to receive all the funding for it, to be able to do this [provide WiFi] for your residents.

Helen

Larry who runs a technology-based activity program for aged care organizations noted the tight budgets of aged care activities programs, which meant it was difficult for them to afford the technology programs his company provides:

I'm not privy to their budgets but in the activity side of things, I think that the people in those teams work very hard to engage a lot of people on a tight budget, so that can be a constraint.

Constraints on staff time were especially problematic in facilitating individual technology-based activities. In aged care homes, group activities are often prioritized over one-on-one activities [6]. Frank, who volunteered in aged care homes, noted that these group activities rarely catered to individual interests and needs:

The organisation has a calendar and it is activities everyday. For instance this morning there is a bus trip that some of them have gone on and there is also an activity where someone will come around and play old Italian songs to them. That’s the limit of stimulation that they get in a day and it’s always done in a group context. One-on-one interaction does not happen. They tend to be herded into groups to do activities. Someone sitting down and actually spending one on one time is very very rare.

The focus on group activities meant that single-user technologies such as VR were sometimes difficult to implement. As noted above, interviewees found VR to be a valuable way of providing enrichment in aged care, as it enabled clients to *leave* the care home and reconnect with past interests. However, using VR in aged care requires careful facilitation and one-on-one support, which is time-consuming for staff who would normally run group activities:

In regard to VR it...does take a bit of one on one time with each customer for them to be able to use it. It’s hard to run that as a group activity because it’s really focused on one person at a time.

Eric

A further challenge is that staff needed time to not only facilitate activities but also learn how to use the technology. This meant that there was an opportunity for external organizations to provide services that aged care staff may not have the time or skills to deliver themselves:

In an aged care environment, it’s a bit harder I think than in independent living space. In care we are finding that there’s so many demands on their time, and the traditional roles they’ve had have been more clinical, a bit more care. They’re great with the emotional side of things, but the skills, the tech skills, we’re having to upskill in that a lot.

Graham

And the other thing is that I think there's great benefits in an external person delivering the service because we've got expertise in how to do that and use the technology, whereas the people in the lifestyle team may be squeezed for time, and I don't know what training they've had with this.

Larry

##### A Need to Select Appropriate Devices and Apps

Many participants used commercially available products, including VR headsets, Skype, and Google Earth. However, these technologies were not designed specifically for older people. Interviewees noted that their designs did not always accommodate the needs of older adults, sometimes creating negative user experiences. Alan noted that a lack of inclusion of older adults in the design and development process led to products of limited value for people in aged care:

Some products aren’t useful because they don’t work very well. There are two reasons, one is they’re targeting a problem that no one has, or they might be targeting an important problem, but they're not well implemented. People are not following established development techniques and involving older adults into the development as much as they should be.

Alan

The content and apps used also needed to be carefully selected to meet the needs of individual clients. Selecting content that did not connect the client with their personal interests could potentially discourage clients or lead to a lack of interest in the activity. Barry noted that it was not possible to implement a *one-size-fits-all* approach when choosing technology-based activities to enrich the lives of individual residents:

Our residents have such a vast variety of likes, dislikes, and there’s so many different factors that come into play as far as that sort of thing is concerned. I'm not sure that there is just one thing that we could put in place that is going to be technology is going to make their day more enriched or anything like that.

Barry

##### Client Challenges

Interviewees spoke of the challenges faced by aged care clients when using new technologies. In some cases, these challenges were attributed to a lack of familiarity with using technology. In others, interviewees noted that technology deployment could be challenging because of frailty and cognitive decline, especially in residential care settings:

I don’t think the technology is the barrier, I think the barrier is the change in residential care. You know 20 years ago our average age of a resident was probably about 75. And that person was scooting about, helping cook meals, do some cleaning and assisting around the place. People are now starting to stay home a lot longer. And...once they come into residential aged care, they’re there simply because they’re at a point where they can't look after themselves...They come in [to aged care] because of issues with dementia or incontinence or things like that. They can't look after themselves or use their hands and their eye sight is going so therefore they can't cook for themselves or clean themselves so therefore using interactive technology becomes a little bit more difficult.

Barry

The interviewees also expressed concerns that the sensory experience of VR may not be appropriate for people with dementia. Barry commented that VR can *displace* people, noting that care is required when introducing such an immersive experience. In line with this observation, Jacquie said that the care home she worked in introduced a VR program in a staged approach:

We decided to first trial residents who still had quite good cognitive ability...But to not necessarily use it straight away on residents living with dementia because it might be a bit too much for them in the beginning. [Interviewer: Why do you think it is too much for people with dementia?]. I think the sensory experience.

Jacquie

For Claire, a further challenge is that clients could experience strong emotional responses when using technology for communication and reminiscence. Although this was often a positive aspect of using technology, there were concerns about the risk of *retraumatizing* clients by “going back to a place or something that might have [a bad] memory.” This concern was not limited to reminiscence activities but extended to situations in which technology was used to facilitate communication with loved ones, which could sometimes be upsetting for clients. Claire carefully monitored these activities:

One time I had an experience where this lady...she was in her late 80s and she had a daughter who was only 60, who developed early-onset dementia. So her daughter was in a nursing home and she was in a nursing home. Her daughter was really bad in comparison to this lady, she was quite advanced...So we used to Skype because she wanted to see her daughter because she couldn’t physically see her...My resident on my side, she got very upset seeing her daughter all the time and she kept saying “it’s not fair...” I actually said to her, “Look, wouldn’t you rather not do this?” and she said “look, despite it being so difficult I do want to do this because I just want to make sure she’s alright.”

Claire

For Claire, this example emphasized how important it was for staff members to be highly skilled in facilitating communication activities for clients. The skills required extended beyond being able to help clients use technology:

If you don’t really understand deep listening and good communication and be able to listen to that and be open to the emotional and spiritual work which the meaning making needs I think you could in some ways do harm.

Claire

Using technology for enrichment then required considerable practical and emotional support, usually provided by care staff and volunteers.

##### Limited Organizational and Staff Support

Interviewees noted that it was crucial to have organizational support, especially support from care staff. Frank, a volunteer who conducted individual VR sessions with aged care residents, said that a lack of staff support was a significant challenge for him:

The biggest challenge I get is pushback from staff. They have their day planned out for this resident. And quite frankly if they can get a resident into [a group] activity it makes their life easier. They are ignorant of the benefit of this sort of stuff.

Frank

In many cases, technologies were incorporated into the activities program offered in residential care homes, and staff running these programs needed to have the skills, capacity, and willingness to use technology. Eric, a technology service provider, said the following:

Probably the biggest challenge is getting a routine around an activities calendar...The VR headset especially, they are a bit fiddly to use at the moment so it takes someone with a bit of specialist knowledge to set it up and have it working in a way that the residents can use it, and because it can be a bit difficult it doesn’t always get incorporated into the activities schedule.

Eric

Ian, also an IT service provider, noted similar constraints that affected his program:

The demographics of people that generally work in aged care did struggle with technology too. Even iPads. We were pretty early on introducing iPads in the organisation and in terms of lifestyle and entertainment type activities. But staff, they struggled with them. Some of them were flat out scared with it, and even just the organisation and support from management were not necessarily there.

Ian

Ian went on to say that there was an “overall inability” within aged care organizations to “do what other people would take as everyday bread and butter IT.” Del suggested that there needed to be a “cultural shift” within organizations, including support from “middle management,” to ensure that technologies were included in the activities program and incorporated into routine care work. Similarly, Graham, a technology provider, noted that “on the ground” support was crucial. Participants claimed that the success of a technology program typically depended on the staff at each aged care home:

The on the ground part is so critical because it can either succeed or fail on that...Basically they [management] can make a decision across a group - so we’ve got one provider that we work closely with and they're rolling it out across six of their homes...From a head office [perspective], they’ve said “yes, we are rolling out,” but then based on each site it is so dependent on the make up of the staff there and turnover as well.

Graham

##### Resistance From Families

In addition to support from staff and management within aged care organizations, interviewees noted that family support was also crucial. Resistance from family members created a barrier that made it difficult for some technologies to be accepted within the aged care setting and limited the benefits the technologies could provide. In the case of robot pets and VR, interviewees noted that family members sometimes saw these as *toys* or *games* that were inappropriate for their loved ones:

We’ve looked at things like the furry seal and so on, but...haven't had the acceptance by residents and I don’t think it’s the residents so much, I think it’s been the family rejection of the things - family members saying, “oh you know my mum or my dad isn’t a child anymore why are you giving them these toys to play with?” Which is sad...but that's something that we have found.

Barry

Occasionally [a challenge] was family members didn't want their loved one to be involved in it. I think that was also that sort of fear thing. “Why would I want my mother playing with a seal?” Or “she doesn't need to look at virtual reality” I think they were scared of the technology.

Ken

In the case of using video calls to connect older people with their family and friends, the connection could only be established with the active participation of family members. In some cases, interviewees observed that although clients were eager to connect with their families, it was not always possible to establish this connection:

I think video calls are as good as anything in connecting people, but you've got to get both sides happy with it, which is why I'm getting frustrated with my client that I want to get connected to his daughter, but I just can't get her phone number...Whether there’s a family feud there, I don’t know.

Ken

A lack of family support was not a universal challenge. Other interviewees described positive experiences, with technology-based activities sometimes providing new opportunities for family members to connect with those living in aged care or learn new things about their loved ones’ lives. However, when family members were not engaged in the programs or when they actively disapproved of the decision to use certain technologies, this created tension that could prevent the ongoing use of technology for enrichment in aged care.

## Discussion

### Principal Findings

This study aimed to understand how technologies are used to enrich the lives of people living in aged care and identify lessons for good practice in this area in the future. The survey and interview findings provide insights into the types of technologies being used in the Australian aged care sector before the advent of the COVID-19 pandemic. These included VR, videoconferencing, and entertainment tools such as YouTube. In terms of perceived value, there were mixed responses for the emerging technologies of VR, robot pets, and social robots. Others such as videoconferencing were viewed more favorably.

Despite mixed views about its perceived value, VR was the most common technology used by our respondents. This finding aligns with the growing research interest in the use of VR in aged care [13,25,30-33]. Studies have demonstrated that VR can be valuable as a calming tool for people with dementia [21], as a tool to support reminiscence in aged care [13], and as a way for people with dementia to enjoy experiences such as attending a concert [34]. Conversely, recent studies have identified usability issues for residents [13,30] and highlighted challenges for staff in implementing VR in aged care [31]. This tension between benefits and challenges was evident in our interviews. On the one hand, we heard compelling stories about the use of VR for virtual travel and reminiscence. On the other hand, interviewees were cautious about the challenges of using VR with aged care clients who are often frail and may experience confusion when confronted with the immersive sensory experiences offered by VR.

Notably, our participants were mostly using off-the-shelf or commercially available technology rather than bespoke apps. For example, videoconferencing provided social connections, tablets and mobile phones provided entertainment, and YouTube and Google Earth provided an easy way to revisit places and connect with past interests. This contrasts with many previous studies evaluating the use of technologies in aged care settings, which have focused on systems designed specifically for use in a particular aged care context [35-37]. Numerous studies have reported evaluations of robot pets, such as Paro, the seal [28], and other social robots designed to provide companionship or lead social activities in aged care [10]. Given this extensive research, it is surprising that of the 20 survey respondents, only 5 (25%) said they had used social or companion robots, and there were limited discussions about robots in the interviews.

Our analysis of interviewees’ stories identified 4 kinds of enrichment experiences or social and emotional benefits for clients. Participants described using a range of technologies that enhanced clients’ social engagement, enabled clients to *leave* the care home, provided opportunities to reconnect with personal interests, and provided entertainment and distraction. All of these involved providing personally meaningful and individual experiences. One of the key constraints of residential aged care is that it can be difficult to provide residents with choice and agency over the activities they are involved in [3]. When used effectively, technology-based activities could help address this need, thereby enhancing agency and control for people living in aged care [33]. However, this may be difficult to achieve in practice. Our findings show that to use technology effectively, care and technology providers need to overcome many challenges, including resource constraints, selecting appropriate devices and apps, client challenges, limited organizational and staff support, and resistance from families.

### Lessons for Deploying Technology for Enrichment in Aged Care

Our interview findings paint a picture of the sociotechnical context that needs to be considered when introducing new technologies into aged care settings, including personal, technological, and social or organizational issues [18]. In the next section, we discuss 3 lessons that can be distilled from our findings, each aligned with an element of this sociotechnical context.

#### Lesson 1: A Person-Centered Care Approach Is Crucial

To create meaningful enrichment experiences, a person-centered care approach is crucial. Aged care activities are often designed in a *one-size-fits-all* model. However, our findings suggest that technology-mediated enrichment activities work best when designed to cater to individual interests and needs. By tailoring activities to meet the needs of individual clients, our participants were able to elicit moments of joy, such as the “whooping and hollering,” which Frank witnessed when he introduced a client to the Formula 1 VR experience.

Notably, our participants spent considerable time getting to know individual clients and understand their needs before introducing technology. For instance, the Formula 1 activity was only introduced after Frank asked the client what his dreams were. Another interviewee, Graham, said that providing personalized connection requires conversation with clients about what they need. Claire also observed that it was crucial for caregivers to listen to and talk with clients to choose technology-based activities that provided personal enrichment.

In addition to meeting individual needs, interviewees were careful to consider their clients’ physical and cognitive health when making decisions about introducing technology to individual clients. For instance, interviewees were cautious about introducing technology to aged care residents who were frail or had advanced dementia, conditions that contribute to the complexity of residential aged care [38]. This caution highlights the gatekeeping role that care providers can have in choosing who will experience a technology-based activity [19]. Gatekeeping can be viewed as a paternalistic approach to care and therefore conflicts with the goal of providing aged care clients with agency and control. However, it may be required to ensure that the technologies provide benefits and do not cause harm. Indeed, understanding an individual’s needs and preferences means knowing when a technology-based activity may not be the best solution [18]. Adopting a person-centered care approach then means accepting that a one-size-fits-all approach is not suitable when deploying technology for enrichment in aged care, despite the efficiency challenges this creates in an organizational setting.

#### Lesson 2: Enrichment Experiences Can Be Created Using Available Technologies, but They Need to Be Carefully Selected and Co-Deployed With Aged Care Clients

Building on the need for a person-centered approach, our findings suggest a need for *co-deployment* of technologies in the care settings in which they are used. We use the term *co-deployment* to refer to collaboration between providers and users when choosing to use, or deploy, particular technologies. This is similar to, but moves beyond, the notion of *co-design*. A study by Wherton et al [39] used the term *co-deployment* to refer to “the mutual shaping of technologies ‘in-use’,” arguing that “older people, their carers, service providers and technology designers must be able to work together to shape technologies and services over time.”

Our findings suggest that in residential care settings, co-deployment starts with choosing to introduce technologies that align with people’s needs, interests, and values. It may not be necessary to design bespoke technologies to meet these needs; instead, caregivers can use available technologies to design technology-mediated enrichment experiences. As noted earlier, many of the experiences our interviewees described were enabled by the use of commercially available technologies rather than bespoke tools or technologies specifically designed for use in aged care. Therefore, our findings suggest that there is a wide array of commercially available tools and apps that can be used to provide social and emotional enrichment in aged care settings. However, these tools need to be carefully selected and deployed as they are not usually designed with aged care clients in mind and may not always meet their needs.

Furthermore, our participants noted that some technologies, despite being designed for use in aged care, may not align with people’s values or address people’s needs for social connection. They were critical of the artificial intelligence devices being used as digital companions, refuting the notion that a conversational agent might provide companionship. This is in contrast to some of the recent research on the use of voice assistants and robot devices that suggests they can provide a sense of companionship [40]. However, recent research also notes that older adults find the concept of digital companionship to be a threat to their sense of dignity; the idea of having a robot pet in the future can be quite confronting [41]. This aligns with our survey responses. As with VR, respondents expressed mixed views about the perceived value of robot pets and social robots. The comments indicated that respondents believed these technologies did not align with human values. They were seen, for instance, to be *spooky* and *child-like* and were not seen to foster *real* personal connections. Despite these comments, however, other research has shown that robot pets, such as Paro, can bring joy and provide a sense of calm for people with dementia [8,42,43]. Indeed, one of our interviewees made a similar observation about Paro, noting its value in providing distraction and reducing agitation.

These divergent views and experiences again emphasize that a one-size-fits-all approach may not be appropriate. Some technology-based activities will work well with some clients because there is a careful match between the person’s values, interests, and needs and the activity being introduced. However, the same technology or activity may not work effectively for others. For instance, the technology may be too difficult or uncomfortable to use for someone who is frail, it might support an activity that is not of interest to the person (eg, a robot that leads a game of bingo will not appeal to some people), or it may be experienced as demeaning. This points to the need both for a person-centered care approach (lesson 1) and a collaborative process of co-deploying technologies that are carefully selected from the array of tools available to meet the needs of individual clients.

#### Lesson 3: The Organizational Context Can Be a Barrier to Effectively Using Individual Technology-Based Activities in Psychosocial Care

Many of the challenges and barriers identified by our interviewees were related to the social or organizational context in which the technologies were being used. Aged care is a complex setting, particularly residential care, where clients are often frail and highly dependent on care [38]. In Australia, the context in which this study was conducted, aged care has been under scrutiny, with a Royal Commission recently highlighting significant neglect, underresourcing, and poor staff-client ratios [44].

This aligns with our interviewees’ observations about the challenges of implementing new technology-based activities when care staff have limited time and resources. Technology-based activities require staff time to learn new skills and introduce activities with care and attention to the needs of individual residents. Resource constraints also affect the funding available to purchase and maintain new technologies, which require significant investment, especially when deployed at a scale for use with multiple clients in a residential facility. Technologies date quickly and may need to be updated or replaced regularly. They also require communication infrastructure, such as wireless networks. Previous research has shown that this can be a barrier; although Wi-Fi is taken for granted in many organizations today, it may still be unavailable in some aged care homes [20]. Similarly, one of our interviewees noted that IT skills taken for granted in other organizations may be absent in the aged care workforce.

Another important element of the organizational context is the norms and routines embedded in an aged care home, with many homes providing a full calendar of organized events on a daily basis [2,45]. These are often group activities. Previous research has shown that staff consider group activities to be a more efficient use of their time than one-on-one activities [5]. This creates a significant barrier for the use of technologies, such as VR and videoconferencing, which typically require one-on-one facilitation by a care provider. Other technologies, such as robot pets, have been used extensively in group settings [8]. However, our study showed divergent views on the value of robots in providing social and emotional enrichment in aged care.

One potential solution to these challenges is to establish a network of volunteers who can work on a one-on-one basis with aged care clients. Such volunteers, however, need to be well supported by staff and management within the care home. Another solution is to use external consultants and organizations that specialize in introducing technology into aged care homes. Some of the interviewees were IT providers from these organizations. Although external consultants may fill an IT skills gap in aged care, there is a need for caution to ensure that such external providers are fully aware of the needs and concerns of aged care clients. Our research showed that combining care and technology requires sensitivity and expertise across multiple domains.

### Limitations and Future Work

First, our study had a small sample size. In particular, we received only 20 responses to our survey, which limits the generalizability of our findings. However, aged care workers are a hard-to-reach group, and those who use technology for client enrichment have specialized expertise. Given the focus on this expertise, a small sample size may be sufficient to provide *information power* [46], especially for in-depth qualitative research.

Second, we focused only on the Australian aged care sector. Care programs in other countries may make use of technology in ways not covered by this study or may have other kinds of constraints not mentioned by our sample. Future work with other samples should be conducted to confirm and extend our findings.

Third, this study did not include the perspectives of older adults themselves or their family members. Previous research has focused on the views and experiences of older adults and family members in evaluation studies of technologies in use in aged care [13,33,35]. In this study, however, we aimed to gain a better understanding of staff experience. In aged care settings, technology-based activities are often facilitated by staff members. Their perspectives and experiences can, therefore, be valuable for understanding what works and does not work well when introducing technology for enrichment in aged care. However, future research in this area should consider the perspectives of all stakeholders, including older adults, family members, and people working in aged care.

Finally, the data for this study were collected before the onset of the COVID-19 pandemic. The use of technology is likely to have expanded following the COVID-19 pandemic, given that aged care homes worldwide had to introduce videoconferencing for family visits and consultations by health specialists [47]. Restrictions brought in to curb the spread of the virus left many older people in aged care more isolated than before [48,49]. This is likely to have increased the need for technologies to maintain social connections. During the COVID-19 pandemic, it has become essential for aged care organizations to use videoconferencing to enable their clients to stay connected to family members and friends [50]. However, it is uncertain whether aged care organizations were prepared to rapidly introduce technology to meet their clients’ social needs during the COVID-19 pandemic. Future work should study the experiences of technology providers during the pandemic to explore what has changed and whether new kinds of technology use have emerged in response to societal restrictions.

### Conclusions

This study showed that a person-centered care approach is required to create personally meaningful and enriching technology-mediated activities in aged care. Although a range of technologies is available, they need to be co-deployed in response to individual needs and interests. However, this requires considerable one-on-one attention and care from staff and volunteers who facilitate the activities, which, given the resource constraints in the aged care sector, may become a barrier to ongoing use. To successfully deploy technologies for enrichment in aged care, significant changes may be required within the aged care sector and within organizations to allow caregivers to facilitate individual technology-based activities to create meaningful enrichment experiences for clients.

## Appendix

Multimedia Appendix 1 Survey questions.

Multimedia Appendix 2 Interview questions for aged care staff.

Multimedia Appendix 3 Interview questions for information technology staff.

## Abbreviations

IT: information technology
VR: virtual reality
