# Supplementary material for: Technology-Mediated Enrichment in Aged Care: Survey and Interview Study
Source: JMIR Aging. 2022 Apr 12;5(2):e31162. doi: 10.2196/31162 (PMC9044160; doi:10.2196/31162)
Supplement: Multimedia Appendix 1 [file aging_v5i2e31162_app1.pdf]

**What kind of aged care do you provide?**	
**What is your role?**	
**What type of technology have you used in aged care for social/activity purposes?**	
**Please provide further details about the technology(ies) you have used)**	
**How did you identify a need for this technology?**	
**In your opinion, how does this technology benefit aged care clients?**	
**What challenges, if any, have you faced in deploying or using this technology in aged care?**	
**Do you believe new technologies should be used in aged care?**	
**Please briefly explain why you think new technologies should or should not be used in aged care.**	
**Please rate on a scale of 1-5 (1=not at all valuable; 5 = highly valuable) how valuable you believe the following technologies are, or could be, when used to support social activities in aged care.**	
**Virtual reality**	
**Robot pets**	
**Social robots**	
**Computer/video games**	
**Social networking tools/systems**	
**Video conferencing tools**	

**Digital storytelling applications**	
**Do you have any comments about why you believe the above technologies are or are not valuable for use in aged care?**	
**What do you believe are the key challenges of using new technologies in social/activity programs in aged care?**	
Other comments?	
**Participate in follow-up interview?**	
