# Supplementary material for: Technology-Mediated Enrichment in Aged Care: Survey and Interview Study
Source: JMIR Aging. 2022 Apr 12;5(2):e31162. doi: 10.2196/31162 (PMC9044160; doi:10.2196/31162)
Supplement: Multimedia Appendix 2 [file aging_v5i2e31162_app2.pdf]

A Study of Technologies used for Enrichment in Aged Care

Interview Questions: Aged Care Staff

1. First, can you tell me a bit about the aged care provider you work for? (e.g., what sort of care do you provide? What is life like for your clients?)

2. What is your role in the organisation?

3. What sort of social activities does your organisation offer for your clients to take part in?

4. Can you tell me about the technology-based activities that you have run? (Why did you introduce these activities? Are you still running them? Why/why not?)

5. How do you believe your clients benefit from taking part in this activity / these activities?

6. Do you believe your clients’ lives were enriched by the activity/activities? Why/why not?

7. Were there any residents who did not benefit or did not enjoy doing the activities? Why do you think they didn’t like it or benefit from it?

8. What challenges did you face when introducing these activities into the care setting? How did you overcome those challenges?

9. Would you recommend this activity to other aged care providers? Why/why not?

10. If you could design the ideal activity for social or emotional enrichment in old age, what would it look like?
