# Supplementary material for: Technology-Mediated Enrichment in Aged Care: Survey and Interview Study
Source: JMIR Aging. 2022 Apr 12;5(2):e31162. doi: 10.2196/31162 (PMC9044160; doi:10.2196/31162)
Supplement: Multimedia Appendix 3 [file aging_v5i2e31162_app3.pdf]

A Study of Technologies used for Enrichment in Aged Care

Interview Questions: Technology Developers / Vendors

1. First, can you tell me a bit about the company you work for? (i.e., what do they do?)

2. What is your role in the organisation?

3. Can you tell me about the technologies you have designed/deployed for use in aged care? (What sort of technologies? What is their purpose?)

4. Why did you start developing/introducing technologies in aged care? (e.g., did you see a need/opportunity for these technologies in aged care?)

5. How do you believe aged care clients benefit from using the technology(ies) you provide?

6. Have there been any instances in which clients did not benefit from or enjoy using the technologies? Why do you think they didn't like it or benefit from it?

7. What challenges did you face when introducing these technologies into aged care settings? How did you overcome those challenges?

8. Would you recommend aged care to other technology developers/vendors? Why/why not?

9. If you could design the ideal technology-based activity for social or emotional enrichment in old age, what would it look like?
